# Identification of anoikis-related subtypes and a risk score prognosis model, the association with TME landscapes and therapeutic responses in hepatocellular carcinoma

**DOI:** 10.3389/fimmu.2025.1602831

**Published:** 2025-06-17

**Authors:** Xiangyu Zhai, Kecheng Li, Hailing Ding, Yanmei Wu, Xinlu Zhang, Hao Zhang, Huaxin Zhou, Chongzhong Liu, Zili Zhang, Bin Jin

**Affiliations:** ^1^ Department of Hepatobiliary Surgery, The Second Hospital of Shandong University, Jinan, China; ^2^ Shandong Province Engineering Research Center for Multidisciplinary Research on Hepatobiliary and Pancreatic Malignant Tumors, Jinan, China; ^3^ Medical Research & Laboratory Diagnostic Center, Jinan Central Hospital Affiliated to Shandong First Medical University, Jinan, China; ^4^ School of Medicine, Qilu Institute of Technology, Jinan, China; ^5^ Institute of Basic Medical Sciences, Shandong Maternal and Child Health Hospital, Jinan, China; ^6^ Basic Medical Research Center, Jinan Central Hospital Affiliated to Shandong First Medical University, Jinan, China; ^7^ Department of General Surgery, The Fourth People’s Hospital of Jinan, Jinan, China

**Keywords:** anoikis, hepatocellular carcinoma, tumor microenvironment, prognostic signature, immunotherapy response

## Abstract

**Introduction:**

Anoikis is a distinct form of programmed cell death, differing from classical apoptosis, and its role in malignant tumor progression, particularly in hepatocellular carcinoma (HCC), remains insufficiently understood. This study aims to elucidate the prognostic significance and therapeutic relevance of anoikis-related genes (ARGs) in HCC.

**Methods:**

We systematically analyzed the expression, mutation, and copy number variation profiles of 27 known ARGs in HCC using public datasets. Unsupervised consensus clustering was performed to classify patients into anoikis subtypes. Weighted Gene Co-expression Network Analysis (WGCNA) identified hub gene modules, and LASSO Cox regression was applied to construct a prognostic risk score model. Correlations between the risk model and clinical outcomes, tumor microenvironment (TME) characteristics, and immunotherapy responses were evaluated. Single-cell RNA-seq and pan-cancer analyses were conducted to explore gene expression across cell types and cancer types. Finally, in vitro experiments were performed to validate the biological function of model genes.

**Results:**

Two distinct anoikis subtypes with differing prognoses and TME features were identified in HCC. A two-gene prognostic model (TTC26 and TPX2) was developed, demonstrating robust performance in predicting patient outcomes. High-risk patients exhibited lower overall survival and distinct immune infiltration profiles. Pan-cancer analysis showed widespread dysregulation of TTC26 and TPX2. In vitro experiments confirmed that TTC26 promotes HCC cell proliferation, migration, and invasion.

**Discussion:**

Our findings reveal that anoikis-related molecular classification is closely linked to HCC prognosis and immune landscape. The established prognostic model has potential clinical utility for risk stratification and treatment guidance. TTC26 may serve as a novel biomarker and therapeutic target in HCC.

## Introduction

Liver cancer is a common malignancy in digestive system, with more than 900,000 new cases and over 830,000 deaths annually worldwide. Among all cancers, liver cancer ranks the sixth and third positions for morbidity and mortality, respectively (1). HCC is the most common pathological type of liver cancer, accounting for approximately 80% of all primary liver cancer cases. Despite advances in surgical techniques, liver transplantation, and the development of targeted therapies and immunotherapies, the prognosis of HCC remains poor, primarily due to late-stage diagnosis, high recurrence rates, and frequent metastasis (2–4), highlighting the need for new molecular markers to predict prognosis and guide personalized treatment strategies.

The tumor microenvironment (TME) plays a crucial role in the development and progression of HCC, influencing both cancer progression and the therapeutic responsiveness, especially immunotherapy (5). TME is a term used to describe the complicated crosstalk of cancer cells with other cellular (eg. immune cells, fibroblasts) and non-cellular components (blood vessels, and extracellular matrix components) within the tumor lesion (6). As one of the most widely-used immunotherapeutics, immune checkpoint inhibitors (ICIs), have shown efficacy in several types of malignant tumors, including HCC. ICI therapies take effect by overcoming the inhibition of classic immune checkpoints, such as PD-1, PD-L1 and CTLA-4 on anti-tumor immune lymphocytes, particularly CD8+ cytotoxic T cells and NK cells, thereby reactivating bodies’ anti-tumor immune response. However, the effectiveness of ICIs is often limited by the immunosuppressive elements of the TME, for instance, some immunosuppressive cells such as TAMs, MDSCs, CAFs, as well as pro-tumor cytokines such as TGF-beta and IL-10 (7). Therefore, a deeper understanding of the TME is crucial to identify patients who are likely to benefit from ICI therapy, allowing for more personalized and effective treatment strategies.

Anoikis is a form of programmed cell death induced by the loss of cell anchorage to the extracellular matrix, which could prevent the aberrant growth and adhesion of cells and is thus essential for the maintenance of body homeostasis (8). In HCC and other malignancies, resistance to anoikis is a critical contributor to tumor progression and metastasis, as it allows cancer cells to survive and disseminate beyond their primary site (9, 10). Anoikis resistance is closely linked to alterations within TME, where factors such as hypoxia, inflammation, and stromal components facilitate the survival of detached cells (11–13). Not surprisingly, multiple anoikis-related genes, such as FAK, bcl-2 and ITGB1 were aberrantly expressed in HCC and associated with patients’ survival (14–16). Besides, the dysregulation of anoikis-related signaling pathways, such as the PI3K/Akt and MAPK pathways, has been implicated in the enhanced invasiveness of HCC tumor cells and could render patients’ resistance to chemo- and immunotherapies (17, 18). Therefore, a deeper understanding of anoikis-related gene signatures would provide novel insights for the prognosis prediction and therapeutic instruction of HCC patients. However, the functional role of anoikis in HCC and its interaction with the immune microenvironment have not yet been systematically elucidated. To date, studies focusing on HCC molecular classification and therapeutic guidance based on anoikis-related features remain limited. Therefore, the present study aims to comprehensively identify key anoikis-related genes through multi-omics data integration, define molecular subtypes of HCC, and construct a robust prognostic risk score model. Furthermore, we evaluate the potential utility of this model in predicting patient survival outcomes, immune infiltration characteristics, and therapeutic responsiveness, thereby providing a theoretical foundation and clinical reference for personalized treatment strategies in HCC.

## Methods

### Data collection

In this study, RNA sequencing (RNA-seq) transcriptomic data and corresponding clinical information for hepatocellular carcinoma (HCC) were obtained from The Cancer Genome Atlas (TCGA)-LIHC database (https://portal.gdc.cancer.gov/). Somatic mutation counts and copy number variation (CNV) data were also sourced from the TCGA database. The 27 anoikis-related genes (ARGs) were retrieved from the Gene Set Enrichment Analysis (GSEA) database (http://www.gsea-msigdb.org/gsea/index.jsp).

### Characteristics of anoikis-related genes

First, we investigated the interaction network among ARGs, including analyses of somatic mutation frequency, genomic loci, and copy number variation (CNV) profiles. The expression patterns of the 27 ARGs were systematically evaluated across different HCC subtypes. Univariate Cox regression analysis was performed using the “coxph” R package to assess the prognostic significance of these genes, and the results were visualized using forest plots.

### Unsupervised consensus clustering

HCC patients were stratified into distinct anoikis-related molecular subtypes based on ARG expression profiles using the “ConsensusClusterPlus” package in R. The robustness of classification was validated through consensus matrix analysis.

### WGCNA analysis

Differentially expressed genes (DEGs) between molecular subtypes were identified using the “limma” R package, with screening criteria set as |log2 Fold Change (FC)| > 1 and false discovery rate (FDR) < 0.01. Subsequently, weighted gene co-expression network analysis (WGCNA) was performed using the “WGCNA” R package. Specifically, the top 5000 genes with the highest median absolute deviation (MAD) were selected for hierarchical clustering. Pearson correlation coefficients were calculated to assess gene similarity. A soft-thresholding power (β = 14) was optimized to construct a scale-free network topology. The adjacency matrix was then transformed into a topological overlap matrix (TOM), and gene modules were identified using a dynamic tree-cutting algorithm with a minimum module size of 30 genes. Pearson correlation analysis was used to evaluate associations between each module and molecular subtypes or clinical traits, and the most significantly associated module was designated as the hub module.

### Prognostic risk model construction and validation

HCC patients were randomly divided into training and validation cohorts at a 1:1 ratio. In the training cohort, univariate Cox regression analysis was first conducted using the “survival” R package to screen prognostically relevant hub genes. Subsequently, the least absolute shrinkage and selection operator (LASSO) Cox regression model was applied via the “glmnet” R package to reduce model overfitting and identify the most robust prognostic markers.

The risk score for each patient was calculated as the sum of the products of normalized expression levels and their corresponding regression coefficients for all selected genes: riskScore = Exp_1_ × Coef_1_ + Exp_2_ × Coef_2_ + ... + Expi × Coefi.In this formula, Exp_i_ denotes the normalized expression level of gene i, Coef_i_ represents the LASSO-derived regression coefficient of gene i, and n is the total number of genes included in the final prognostic signature.

Patients in both the training and validation cohorts were then stratified into high-risk and low-risk groups based on the median risk score calculated from the training cohort.

### Nomogram development

A nomogram integrating risk scores and clinical parameters was constructed using the “rms” R package to predict survival probabilities at specific timepoints. Calibration curves evaluated prediction accuracy, while ROC curves and AUC values quantified discriminative power. Decision curve analysis (DCA) compared net benefits among nomogram, risk score, and clinical data alone.

### Functional enrichment analysis

Kyoto Encyclopedia of Genes and Genomes (KEGG) pathway enrichment was performed via GSEA. Terms with |normalized enrichment score (NES)| > 1 and FDR < 0.05 were deemed significant.

### Tumor microenvironment and immunotherapy response

Immune/stromal scores were computed using ESTIMATE to quantify TME components. Immune cell infiltration levels were assessed via ssGSEA based on Charoentong-defined marker genes. The prognostic model was applied to the IMvigor210 cohort (anti-PD-L1-treated patients) to compare risk scores between responders/non-responders and survival outcomes.

### Single-cell expression of ARGs

The Tumor Immune Single-Cell Hub (TISCH) database, comprising single-cell transcriptomic profiles of ~2 million cells from 27 cancer types, was utilized to delineate ARG expression across TME cell subtypes in HCC.

### Pan-cancer analysis

Tumor mutation burden (TMB), microsatellite instability (MSI), and CD274 (PD-L1) levels were compared across 33 cancers. Correlations between risk scores, TME features, and stemness indices were evaluated.

### Cell culture

Human hepatocellular carcinoma cell lines Huh7 and Hep3B were obtained from the Cell Bank of the Chinese Academy of Sciences (Shanghai, China). Cells were cultured in high-glucose Dulbecco’s Modified Eagle Medium (DMEM, Gibco, C11995500BT) supplemented with 10% fetal bovine serum (FBS, Procell, 164210-500) and 1% penicillin-streptomycin solution (Gibco, 15140122). Cultures were maintained at 37 °C in a humidified incubator with 5% CO_2_. Hypoxic conditions (1% O_2_, 5% CO_2_, 94% N_2_) were established using a tri-gas incubator (Eppendorf Galaxy 48 R), and the culture duration under hypoxia was consistent with normoxic controls unless otherwise specified. Cell line identity was verified by short tandem repeat (STR) profiling, and mycoplasma contamination was routinely tested using PCR-based MycoBlue™ Mycoplasma Detection Kit (Vazyme, D101-01).

### RNA analysis and qPCR

Total RNA was extracted using TRIzol Reagent (Vazyme, R401-01) following the manufacturer’s protocol, including chloroform-mediated phase separation and isopropanol precipitation. RNA purity and concentration were assessed using a NanoDrop 2000 spectrophotometer (Thermo Fisher). Subsequently, 1 μg of total RNA was reverse-transcribed into cDNA using the HiScript II Q Select RT SuperMix for qPCR (+gDNA wiper) (Vazyme, R233-01) according to the recommended protocol (20 μL reaction volume).

Quantitative PCR was performed using Taq Pro Universal SYBR qPCR Master Mix (Vazyme, Q712-02) in a 20 μL reaction system on the QuantStudio 5 Real-Time PCR System (Applied Biosystems). The thermal cycling conditions were as follows: initial denaturation at 95 °C for 30 s, followed by 40 cycles of denaturation at 95 °C for 10 s and annealing/extension at 60 °C for 30 s. Melt curve analysis was performed to confirm amplification specificity. Each sample was analyzed in triplicate. Gene expression levels were normalized to GAPDH and calculated using the 2^−ΔΔCt method.

### Protein immunoblotting

Proteins extracted with RIPA buffer (Beyotime) containing inhibitors were quantified by BCA assay. Equal protein amounts were separated via SDS-PAGE and transferred to PVDF membranes. After blocking (5% milk), membranes were probed with primary antibodies overnight (4°C), followed by HRP-conjugated secondary antibodies. ECL substrate (Tanon) enabled band visualization, with quantification performed using ImageJ.

### Proliferation and clonogenic evaluation

CCK-8 assays involved seeding 2×10³ cells/well (96-well plates) with absorbance (450 nm) recorded at 24–72 h post-reagent addition. For colony formation, 500 cells/well (6-well plates) were cultured for 10–14 days, followed by fixation (4% PFA), crystal violet staining, and manual colony counting.

### EdU and migration/invasion assays

EdU incorporation was assessed using BeyoClick™ kits, followed by fixation (4% PFA). Photograph the labeled cells under a fluorescence microscope. Transwell chambers (Corning) coated with (invasion) or without (migration) Matrigel received 5×10^4^ serum-starved cells. Migrated cells were stained and counted after 36 h.

### Wound healing analysis

Confluent monolayers in 6-well plates were scratched using sterile tips. After PBS washing, serum-free medium was added, and wound closure was tracked at 0/24/48 h via phase-contrast microscopy (Nikon). Migration rates were quantified by ImageJ-based analysis of wound area reduction.

### Statistical analysis

All analyses were conducted in R 4.1.1. Wilcoxon and Kruskal-Wallis tests compared two or multiple non-parametric groups, respectively. T-tests and ANOVA were used for parametric data. Significance thresholds: *P < 0.05; **P < 0.01; ***P < 0.001.

## Results

### Genetic alterations of anoikis-related genes in HCC

**Figure 1:**
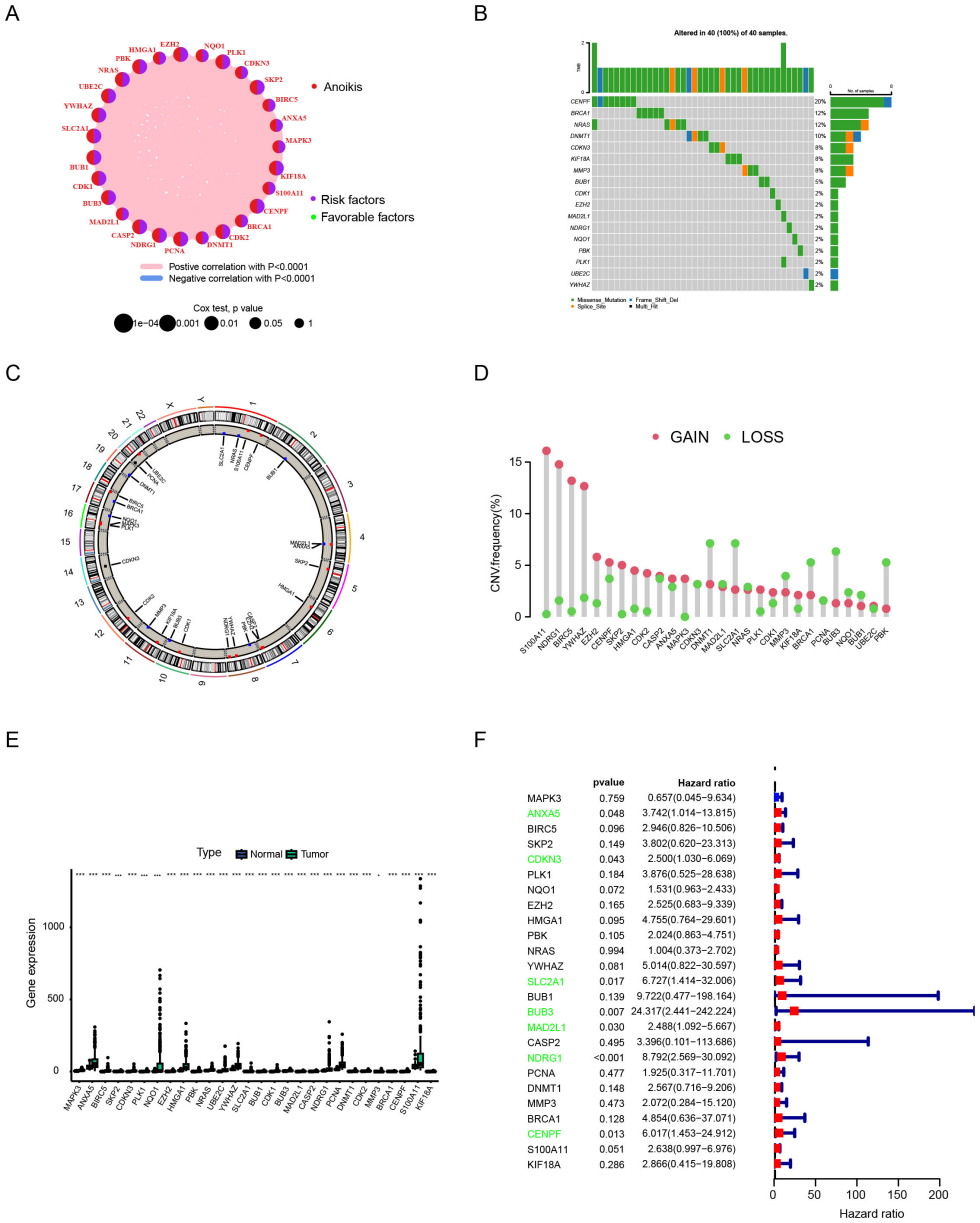
Analytical approaches for characterizing anoikis-related genetic alterations in hepatocellular carcinoma. **(A)** Network analysis of gene-prognosis interactions. **(B)** Somatic mutation frequency profiling. **(C)** Chromosomal localization mapping. **(D)** Copy number variation (CNV) landscape. **(E)** Differential gene expression analysis between tumor and normal tissues. **(F)** Survival correlation assessment using integrated GEO datasets. Data are expressed as mean ± SEM. Statistical significance was determined by a two-tailed Student’s t-test. *p < 0.05, ***p < 0.001.

A total of 27 anoikis - related genes were included in this study. A network diagram was used to visualize the comprehensive and intricate relationship between anoikis - related genes and the prognosis of HCC (**Figure 1A**). We investigated the somatic mutation rates of the 27 anoikis - related genes in HCC (**Figure 1B**). Among them, CENPF had the highest mutation rate (up to 20%), while the mutation rates of other genes were relatively low. **Figure 1C** shows the specific chromosomal locations of the anoikis - related genes. In addition, the copy number variation (CNV) of the 27 anoikis - related genes was analyzed. As shown in **Figure 1D**, CNV was prevalent. S100A11, NDRG1, BIRC5, YWHAZ, CENPF, SKP2, HMGA1, CDK2, MAPK3, and PLK1 showed widespread CNV increases, while DNMT1, SLC2A1, MMP3, BRCA1, BUB3, and PBK showed CNV deletions. Compared with normal tissues, most of the anoikis - related genes were significantly up - regulated in HCC tissues (**Figure 1E**). When we explored the impact of the 27 anoikis - related genes on the overall survival (OS) of the comprehensive GEO dataset, we found that the expression of ANXA5, CDKN3, SLC2A1, BUB3, MAD2L1, NDRG1, and CENPF was statistically correlated with the OS of HCC patients (**Figure 1F**).

### Identification of anoikis subtypes

**Figure 2:**
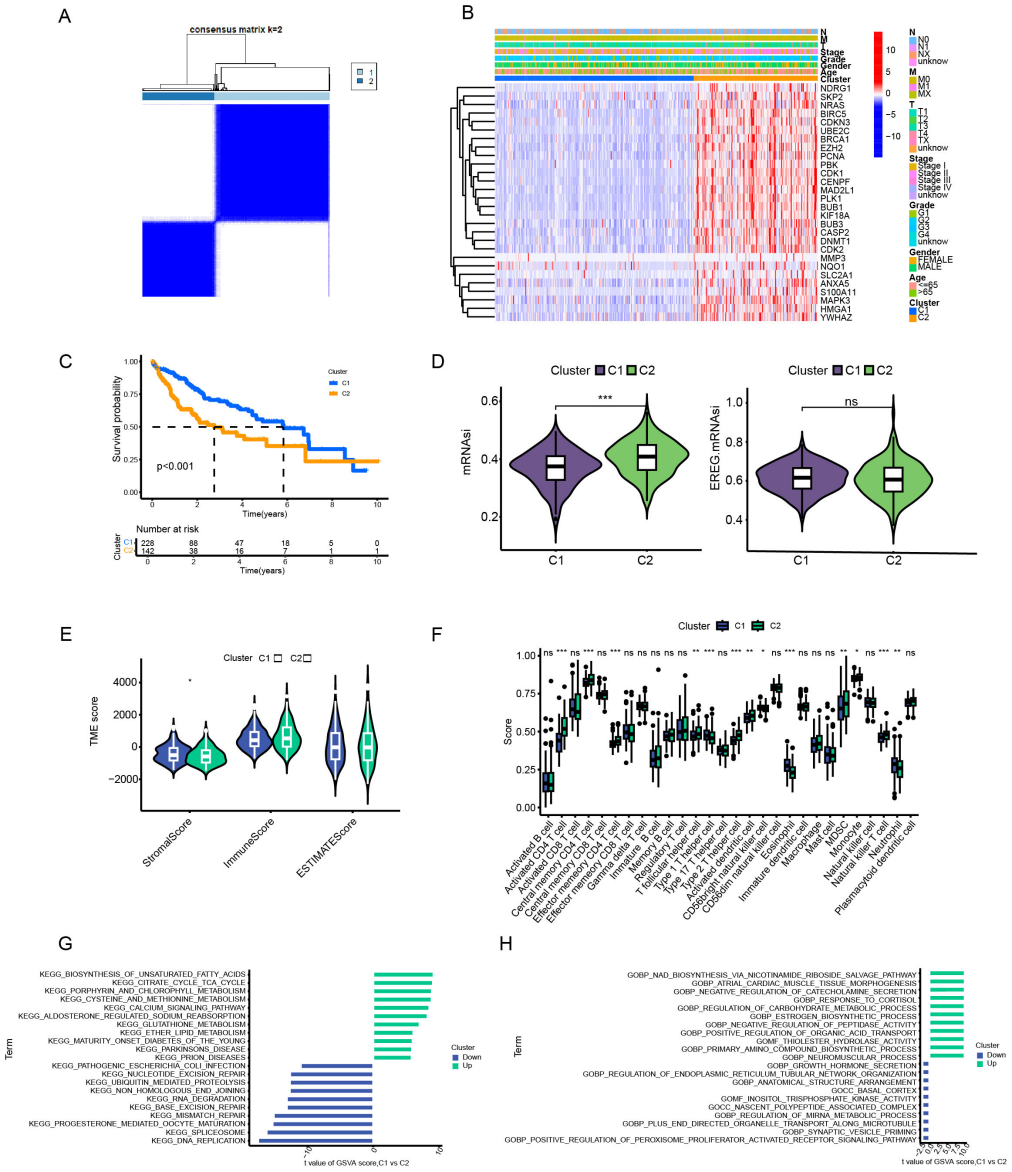
Analytical framework for anoikis-related molecular subtyping in hepatocellular carcinoma. **(A)** Consensus clustering analysis. **(B)** Gene expression profiling by subtype. **(C)** Survival probability assessment (Kaplan-Meier). **(D)** Tumor stemness evaluation (mRNAsi scoring). **(E)** Tumor microenvironment characterization (ESTIMATE algorithm). **(F)** Immune cell infiltration analysis. **(G, H)** Pathway enrichment analysis (GSVA) based on KEGG/GO gene sets. Data are expressed as mean ± SEM. Statistical significance was determined by a two-tailed Student’s t-test. *p < 0.05, **p < 0.01, ***p < 0.001, ns: not significant.

We used the consensus clustering algorithm to divide 370 HCC patients into two subtypes, named C1 and C2 (**Figure 2A**). According to the heatmap in **Figure 2**, all 27 anoikis - related genes had higher expression levels in the C2 subtype. The results of the Kaplan - Meier analysis showed that the overall survival rate of patients in the C1 subtype was significantly better than that in the C2 subtype (p < 0.001) (**Figure 2C**). According to the mRNAsi analysis, the C2 subtype showed higher stemness compared with the C1 subtype, indicating stronger invasiveness of its tumor cells (**Figure 2D**). We also calculated the immune score and stromal score based on the ESTIMATE algorithm. The stromal score of C2 was lower than that of C1, while there was no significant difference in the immune score and tumor purity between the two (**Figure 2E**). Regarding the differences in the tumor microenvironment (TME) between the two subtypes, we observed that C2 was rich in the infiltration of several immune cells, such as Activated CD4 T cell, Central memory CD4 T cell, Effector memory CD4 T cell, T follicular helper cell, Type 1 T helper cell, etc. (**Figure 2F**). In addition, we conducted a Gene Set Variation Analysis (GSVA) on the two subtypes of patients based on the Kyoto Encyclopedia of Genes and Genomes (KEGG) and Gene Ontology (GO) gene sets. In the KEGG gene set, entries such as Unsaturated Fatty Acids, Citrate Cycle TCA Cycle, Porphyrin and Chlorophyll Metabolism, and Cysteine and Methionine Metabolism were more highly enriched in C1, suggesting higher metabolic activity. While entries such as DNA Replication, Spliceosome, Progesterone Mediated Oocyte Maturation, and Mismatch Repair were more active in C2, indicating stronger proliferative capacity of its tumor cells (**Figure 2G**). In the GO gene set, similarly, metabolism - related entries such as response to cortisol and regulation of carbohydrate metabolic process had a higher enrichment level in the C1 subtype, while entries related to tumorigenesis and development such as ER tubular organization and PPAR signaling pathway were more active in the C2 subtype (**Figure 2H**).

### Construction and validation of risk scores

**Figure 3:**
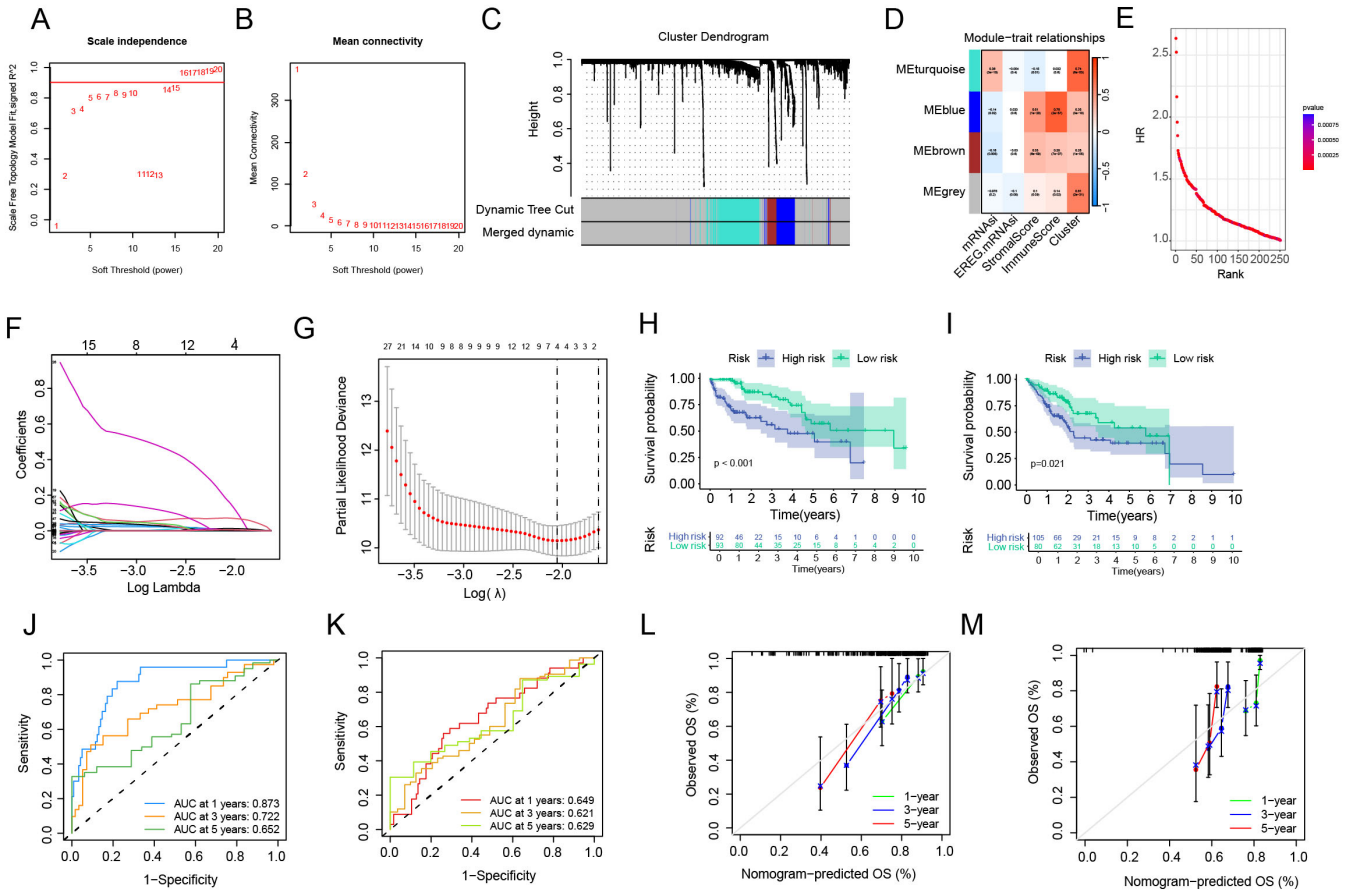
Construction and validation of the anoikis-related prognostic risk model. **(A, B)** Identification of the optimal soft-thresholding power. **(C)** Differential gene expression analysis between molecular subtypes. **(D)** Weighted gene co-expression network (WGCNA) module-trait correlation analysis. **(E)** Univariate Cox regression for preliminary prognostic gene screening. **(F, G)** LASSO regression-based gene selection and model optimization. **(H, I)** Survival probability stratification (Kaplan-Meier curves). **(J, K)** Predictive performance evaluation (ROC curves). **(L, M)** Model calibration analysis.

In order to obtain the key gene modules most closely related to clinical characteristics, we first used the limma package to obtain the differentially expressed genes (DEGs) between the C1 and C2 subtypes. Under the criteria of |logFC| > 1.0, FDR < 0.05, a total of 1755 DEGs were obtained. Next, we performed Weighted Gene Co-expression Network Analysis (WGCNA) on the above- mentioned genes and determined the optimal soft-thresholding power as 14 to further identify the gene modules most closely associated with stemness and tumor microenvironment (TME) scores (**Figures 3A–C**). As shown in **Figure 3D**, the MEblue module exhibited the strongest correlation with clinical traits. Subsequently, all HCC patients were divided into a training set and a validation set at a ratio of 1:1. In the training set, a univariate Cox regression algorithm was used to initially obtain 251 genes related to HCC prognosis, and the distribution curve of their hazard ratios (HRs) is shown in **Figure 3E**. Next, we attempted to use the LASSO algorithm to eliminate over - fitting among genes, and finally identified two genes, TTC26 and TPX2 (**Figures 3F, G**), for constructing a risk - scoring model. Subsequently, the model was applied to the validation set, and patients were divided into high - risk and low - risk groups using the same criteria as the training set. According to the survival curves, the overall survival (OS) of patients in the high - risk group was significantly shorter than that of patients in the low - risk group (**Figures 3H, I**). The receiver operating characteristic (ROC) curves confirmed the good predictive performance of the risk score for the 1 - year, 3 - year, and 5 - year survival rates of HCC patients, as their area under the curve (AUC) values were all over 0.6 (**Figures 3J, K**). In addition, calibration curves were drawn to analyze the gap between the predicted values of the risk score for the survival rates at different time points and the actual observed situations. As shown in **Figures 3L, M**, the predicted values were highly consistent with the actual situations. The above results indicate that our prognostic model has good discriminatory and predictive abilities for the survival outcomes of HCC patients.

### Correlation of risk score with clinicopathological features of HCC

**Figure 4:**
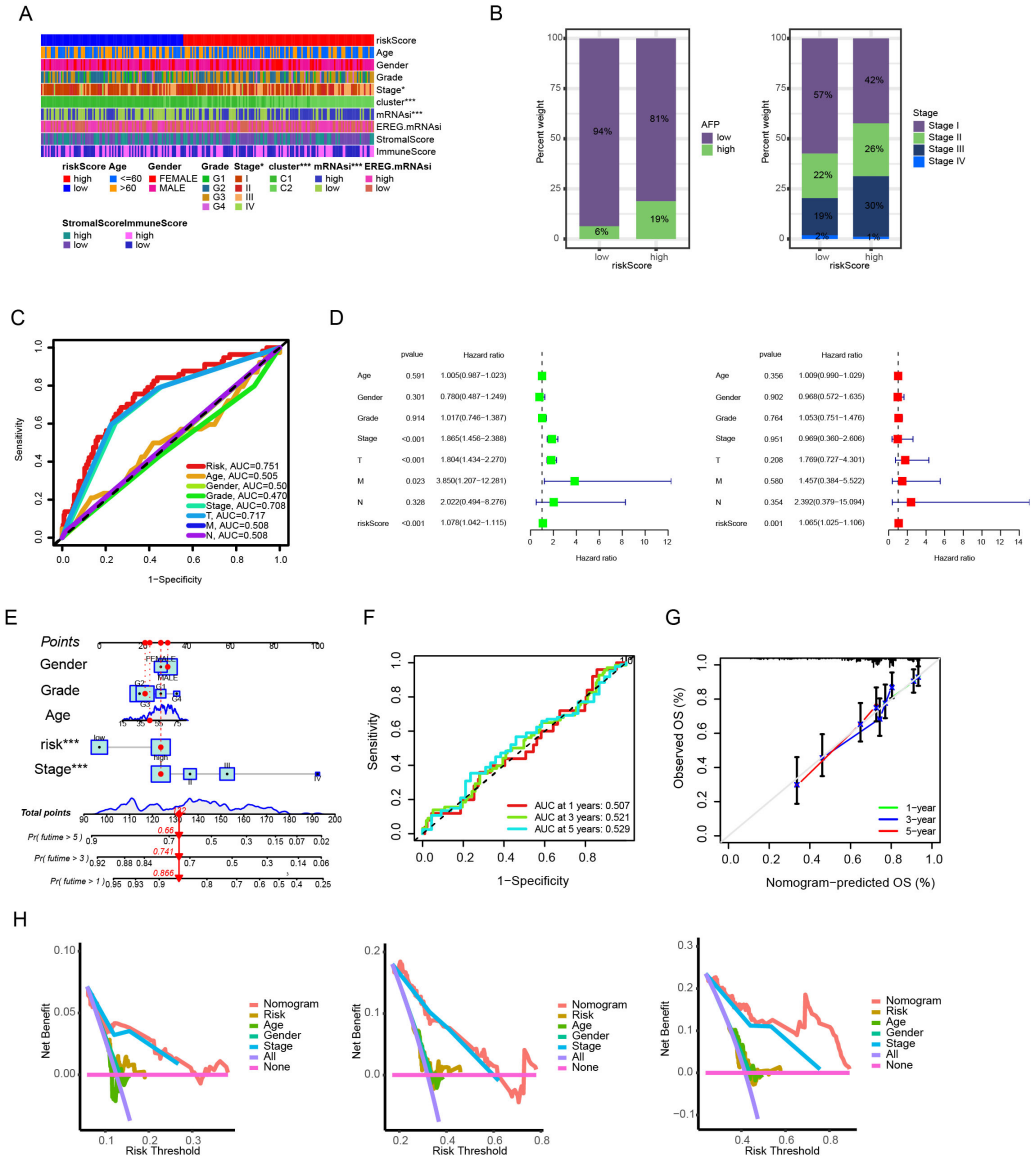
Clinical correlation and prognostic utility of the anoikis-based risk model. **(A)** Clinicopathological feature stratification by risk groups. **(B)** Distribution patterns of AFP levels and TNM stages. **(C)** Prognostic predictive performance comparison across variables. **(D)** Univariate and multivariate Cox regression analyses for prognostic independence. **(E)** Nomogram integrating risk scores and clinical parameters. **(F)** Time-dependent predictive accuracy evaluation (ROC curves). **(G)** Model calibration assessment. **(H)** Clinical benefit analysis via decision curve methodology.

We further analyzed the correlation between the prognostic model and the clinicopathological features of HCC. As shown in **Figure 4A**, there were significant differences in the stage between HCC patients in the high-risk and low-risk groups. Specifically, HCC patients with higher AFP levels and advanced TNM stages (stage III and IV) were more frequently distributed in the high - risk group (**Figure 4B**). Compared with other clinicopathological features, the risk score had a higher predictive efficacy for the prognosis of HCC patients (**Figure 4C**). Univariate and multivariate COX analyses indicated that the risk score could serve as an independent factor for predicting the prognosis of HCC patients (**Figure 4D**). To strengthen the connection between the model and clinical practice and further improve its predictive effect on the prognosis of HCC patients, this study further constructed a nomogram that incorporated the risk score and other clinical factors (**Figure 4E**). According to the ROC curves, the nomogram demonstrated good predictive efficacy for the 1 - year, 3 - year, and 5 - year survival rates (**Figure 4F**). Based on the calibration curves, there was a high degree of consistency between the predicted values of the nomogram and the actual observed situations, suggesting good predictive accuracy (**Figure 4G**). Additionally, the decision curve analysis (DCA) showed that the nomogram had a higher net benefit rate for prognostic prediction overall compared to the simple risk score and clinical data alone (**Figure 4H**).

### Correlation of risk score with TME characteristics in HCC

**Figure 5:**
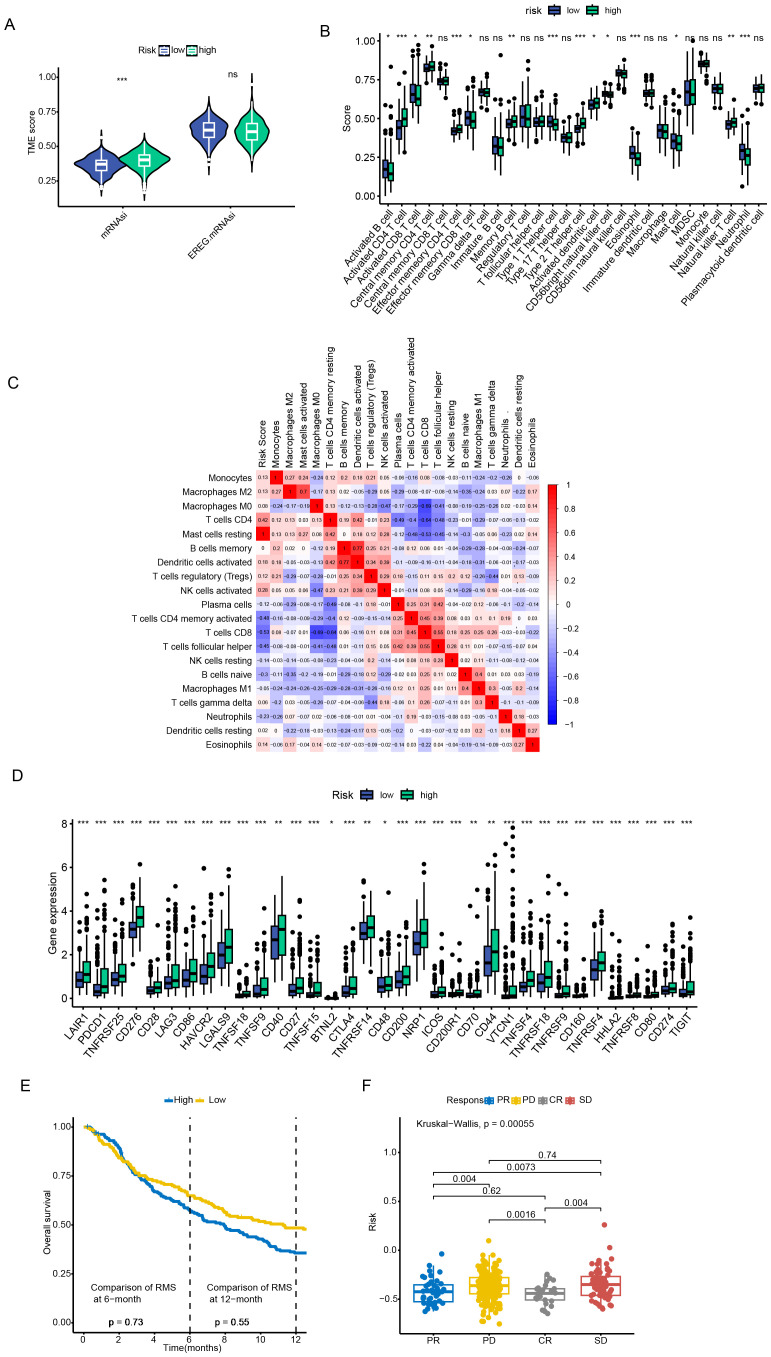
Tumor microenvironment characterization and immunotherapy response evaluation. **(A)** Stromal and immune score comparison between risk groups. **(B, C)** Immune cell infiltration profiling via ssGSEA algorithm. **(D)** Immune checkpoint genes were significantly upregulated in patients in the high-risk group. **(E, F)** Survival outcomes stratification by risk groups in ICI-treated cohorts. Data are expressed as mean ± SEM. Statistical significance was determined by a two-tailed Student’s t-test. *p < 0.05, **p < 0.01, ***p < 0.001, ns: not significant.

Regarding the TME scores, the stromal score was higher in patients of the low - risk group, while there was no significant difference in the immune scores between the two groups of patients (**Figure 5A**). In addition, the ssGSEA algorithm analysis revealed significant differences in the contents of different types of immune cells between the high - risk and low - risk groups. For example, the contents of CD4+ effector and memory T cells were higher in the high - risk group, while CD8+ effector and memory T cells were more inclined to infiltrate into the TME of patients in the low - risk group. Moreover, the two types of immunosuppressive cells, MDSC and Th2 cells, had a higher degree of infiltration in the high - risk group, while Th1 cells with anti - tumor activity had an advantage in content in the low - risk group (**Figures 5B, C**). Given the differences in TME characteristics between the two groups of patients, this paper further evaluated the predictive value of the prognostic model for the efficacy of immunotherapy. First, multipile immune checkpoint genes were differentially expressed between high and low risk groups (**Figure 5D**), then we applied the prognostic model to the IMvigor210 cohort, compared the differences in risk scores among patient groups with different treatment responses, and examined the survival of patients in the high-risk and low-risk groups after receiving ICI treatment. As shown in **Figures 5E, F**, the risk scores of patients with CR/PR were significantly lower than those of patients with SD/PD. The overall survival (OS) of patients in the low - risk group after receiving ICI treatment was significantly better than that of patients in the high - risk group. These results support a greater likelihood of patients in the low - risk group benefiting from ICI therapy.

### Single-cell analysis of prognostic models

**Figure 6:**
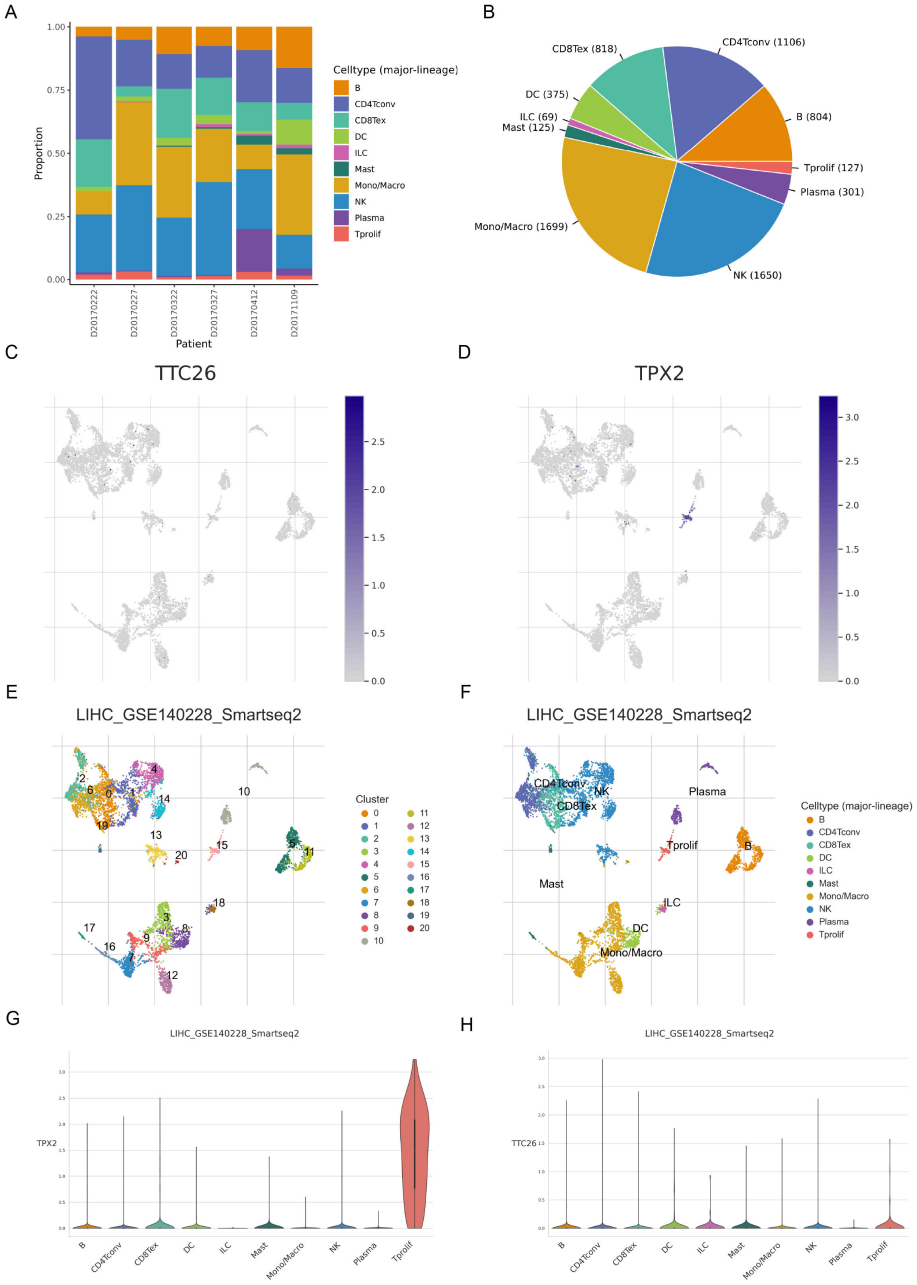
Single-cell transcriptomic profiling of anoikis-related genes in hepatocellular carcinoma. **(A–D)** Single-cell clustering and cell type annotation using the TISCH database (GSE140228 dataset). **(E–H)** Cell type-specific expression patterns of ARGs in the tumor microenvironment.

To analyze the expression of ARGs in different types of cells within the TME, we analyzed the single - cell sequencing data of HCC from GSE140228 through the TISCH database. A total of 20 cell populations and 10 cell types were identified (**Figures 6A–D**). Among them, TPX2 was mainly expressed in proliferative T cells, while TTC26 had a relatively low expression level in all cell types (**Figures 6E–H**).

### Pan-cancer and drug sensitivity analysis of model genes

**Figure 7:**
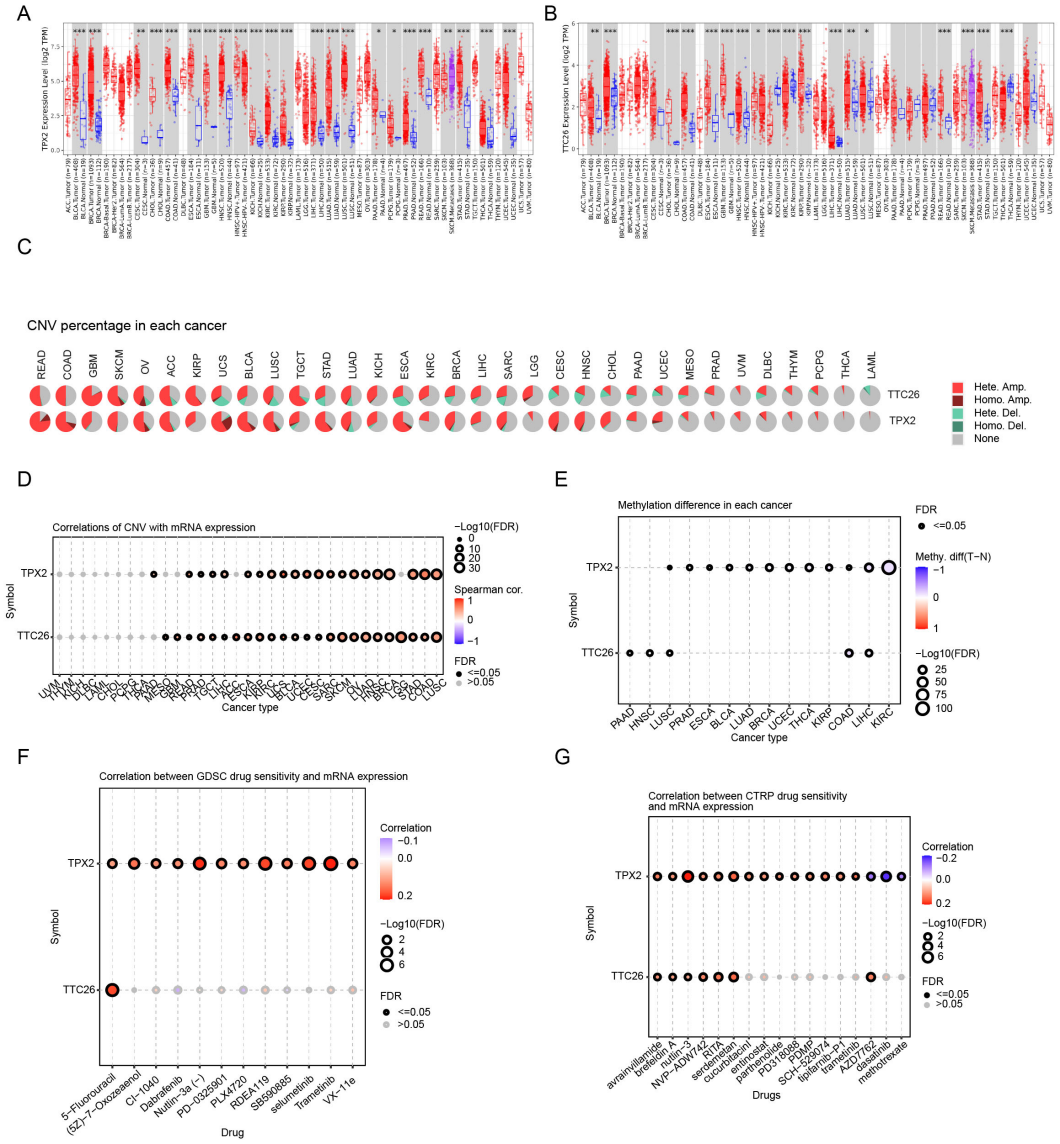
Pan-cancer genomic and pharmacogenomic characterization of prognostic genes. **(A, B)** Pan-cancer expression profiling of TPX2 and TTC26. **(C, D)** CNV association analysis across cancer types. **(E)** Association with DNA methylation levels. **(F, G)** Drug sensitivity correlation analysis (GDSC and CTRP databases). Data are expressed as mean ± SEM. Statistical significance was determined by a two-tailed Student’s t-test. *p < 0.05, **p < 0.01, ***p < 0.001.

We further investigated genetic alterations of the prognostic model genes across pan-cancer. TPX2 and TTC26 are mainly significantly upregulated in a variety of malignant tumors. (**Figures 7A, B**). Both genes displayed positive correlations between expression levels and copy number variation (CNV), with heterozygous amplification (het.amp) being the most frequent CNV subtype (**Figures 7C, D**). Additionally, TPX2 and TTC26 expression inversely correlated with DNA methylation levels (**Figure 7E**), suggesting that CNV alterations and epigenetic modulation may synergistically drive their dysregulation in cancer.

To assess the clinical utility of these genes in therapeutic decision-making, we analyzed their associations with drug sensitivity using the GDSC and CTRP databases. Strikingly, both TPX2 and TTC26 demonstrated positive correlations with IC50 values for the majority of chemotherapeutic and targeted agents (**Figures 7F, G**). These findings imply that tumors with low TPX2 or TTC26 expression may exhibit enhanced responsiveness to conventional chemotherapy and targeted therapies.

### TTC26 is identified as a key driver of HCC progression

**Figure 8:**
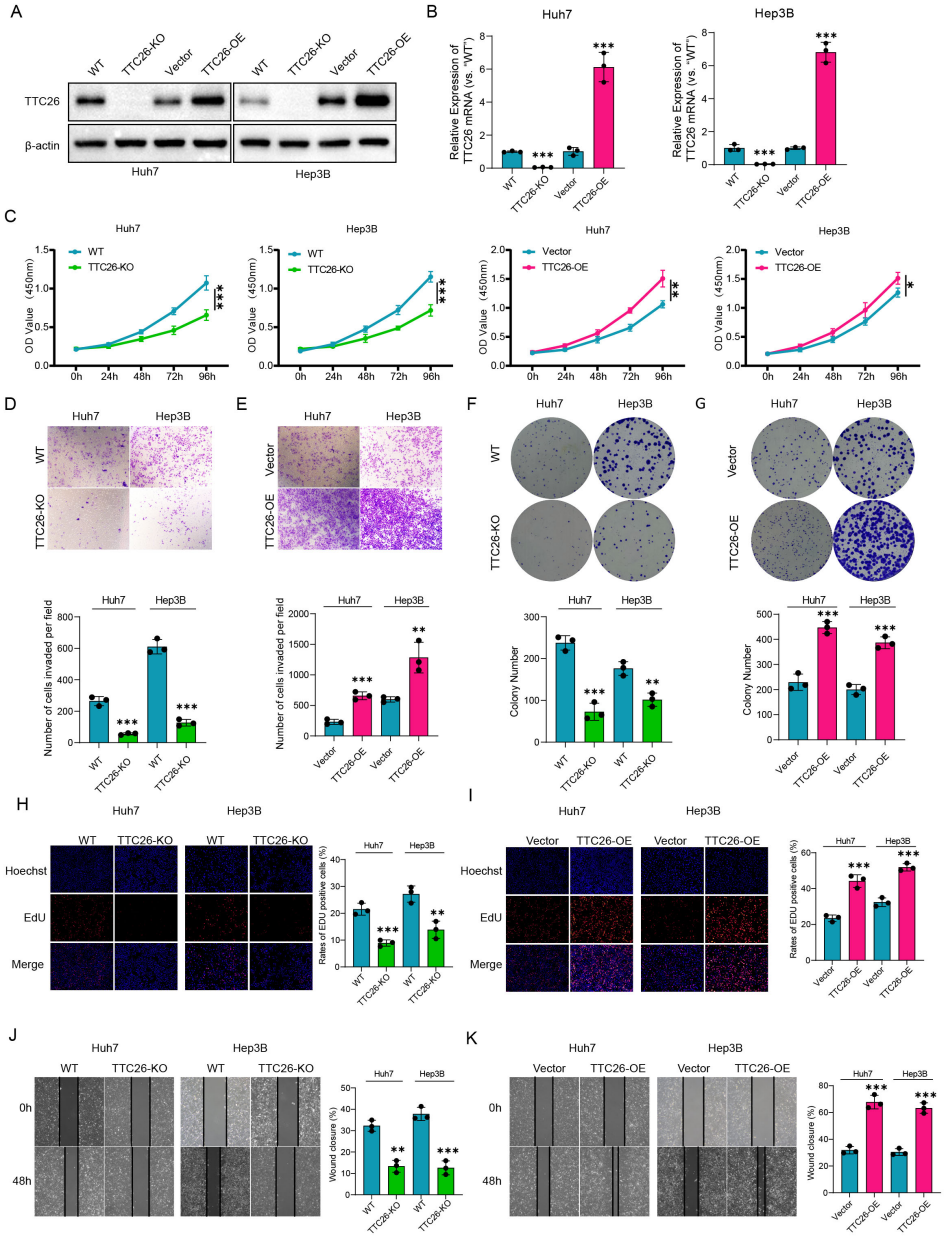
Experimental validation and functional characterization of TTC26 in HCC models. **(A)** Western blot validation of genetic modifications. **(B)** qRT-PCR confirmation of TTC26 expression levels. **(C)** Cell proliferation assessment (CCK-8 assay). **(D, E)** Invasion capacity evaluation (Transwell assay). **(F, G)** Clonogenic potential analysis. **(H, I)** Proliferative activity quantification (EdU assay). **(J, K)** Migration capability assessment (scratch wound healing assay). Data are expressed as mean ± SEM of three independent experiments. Significance was determined by two-tailed Student’s t-test. *p < 0.05, **p < 0.01, ***p < 0.001.

Western blot analysis demonstrated that the protein expression in the constructed TTC26 knockout and overexpression cell models confirmed the successful establishment of the corresponding cell lines.(**Figure 8A**). TTC26 knockout/overexpression models were rigorously validated through qRT-PCR (**Figure 8B**). Comprehensive functional characterization revealed TTC26’s critical role in modulating HCC malignancy through oncogenic mechanisms: TTC26 overexpression enhanced proliferative capacity as evidenced by CCK-8 assays, while knockdown models showed proliferation reduction (**Figure 8C**). Consistent with the above findings, Transwell assays demonstrated that TTC26 overexpression significantly enhanced cellular invasion, while knockout treatment markedly reduced invasive capacity (**Figures 8D, E**). Clonogenic assays revealed that TTC26-overexpressing cells exhibited a substantial increase in colony-forming ability, whereas the knockout group showed a significant decrease in colony formation (**Figures 8F, G**). In addition to the CCK-8 assay confirming alterations in proliferative capacity, EdU assays further validated TTC26’s functional role in tumor cells: TTC26 overexpression significantly enhanced proliferative activity, while the knockout group displayed markedly reduced proliferation (**Figures 8H, I**). Regarding cellular migration, scratch assays corroborated this migratory phenotype, with TTC26-overexpressing cells demonstrating accelerated wound closure compared to delayed healing in knockout models (**Figures 8J, K**). Statistical confirmation of TTC26’s tumor-promoting effects was consistent across all experimental paradigms (P<0.05). Data represent mean ± SEM from triplicate experiments (n=3). Significance thresholds: *P<0.05, P<0.01, *P<0.001.

## Discussion

Hepatocellular carcinoma (HCC) is a significant cancer threatening human health, and the exploration of its molecular markers has been a research focus. Apoptosis is a classic form of programmed cell death. During cancer development, tumor cells could gain resistance to apoptosis, which is regarded as a hallmark of cancer (19). Inducing apoptosis in cancer cells has emerged as an effective anti-tumor therapeutic strategy (20). Anoikis is a specific form of apoptosis that occurs when cells lose adherence or improperly adhere to the extracellular matrix. Tumor cells can resist anoikis through various strategies, such as regulating the expression of adhesion molecules and undergoing epithelial-mesenchymal transition (21). This study aimed to comprehensively analyze the genetic alterations of anoikis-related genes in HCC and utilize these genes for molecular subtyping of HCC patients. Additionally, we constructed an anoikis-related risk score prognostic model to quantify the survival risk of HCC patients, providing novel insights into the prognosis prediction and comprehensive management of HCC.

Our study first revealed the global transcriptional alterations of anoikis-related genes (ARGs) in HCC patients. Next, based on the expression patterns of ARGs, we used an unsupervised consensus clustering method to divide TCGA-HCC patients into two anoikis subtypes, termed as C1 and C2. This subtyping system showed outstanding performance regarding the differentiation of prognosis, functional enrichment, and tumor microenvironment (TME) characteristics for HCC patients. To enhance the clinical utility of anoikis-based subtyping, we further developed a simplified anoikis score prognostic model following the protocols below. First, differentially expressed genes (DEGs) between two subtypes were identified, followed by weighted gene co-expression network analysis (WGCNA) to select gene modules most strongly associated with subtypes, TME scores, and cancer stemness. Next, univariate Cox regression analysis was conducted to identify prognostic-related DEGs. Patients were then divided into training and validation cohorts in a 1:1 ratio, and a two-gene model was constructed in the training set using LASSO Cox regression analysis, with its predictive efficacy later verified in the validation sohort. Survival curves showed that patients in the low-risk group had significantly better overall survival (OS) than those in the high-risk group. The prognostic model demonstrated good predictive efficacy for 1-, 3-, and 5-year survival rates according to ROC curves. A nomogram integrating the risk score with other prognostic clinical factors enabled more accurate patient prognosis predictions.

In our study, HCC patients in the C2 subtype and high-risk group demonstrated higher tumor stem cell scores and biological functions mainly related to cell proliferation, indicating a higher degree of tumor malignancy and poorer prognosis. Regarding the TME, the stroma score of the C1 subtype was significantly higher than that of the C2 subtype, while no significant differences in immune scores were observed among patients with different subtypes and risk levels. Regarding the specific immune cell subtypes, various antitumor lymphocytes displayed distinct distributions between the two subtypes, as well as two risk groups. For instance, CD4+ effector and memory T cells were more abundant in the C2 subtype and high-risk group, whereas CD8^+^ effector and memory T cells preferred to infiltrate in the TME of low-risk patients. In addition, two types of pro-tumor immune cells, MDSC and Th2 cells, had a higher degree of infiltration in C2 subtype and/or high-risk group, while Th1 cells with anti-tumor activity were more dominant in C1 subtype and low-risk group. MDSCs, defined as pathologically activated neutrophils and monocytes, can inhibit the function of antitumor lymphocytes by secreting cytokines like TGF-β and IL-10 (22). Additionally, the pro-tumor effects of MDSCs relate to their induction of cancer cell stemness and epithelial-mesenchymal transition (23). T helper cells, based on secreted cytokines, are categorized into Th1, Th2, and Th17 types. Th1 cells enhance antitumor immune responses by secreting interferon-γ (IFN-γ), while Th2 cells create an immunosuppressive TME by secreting IL-4 and IL-10, weakening cell-mediated immune responses and promoting tumor immune evasion (24). Th17 cells secrete IL-17, influencing antitumor immunity in a more complex manner. On the one hand, IL-17 promotes dendritic cell activation, thus enhancing the antitumor activity of CD8 T cells. On the other hand, IL-17 could implement pro-tumor functions by activating oncogenic pathways like MAPK and STAT3 in tumor cells or via the interactions with other TME components (25). As expected, MDSCs and Th2 cells with immunosuppressive features were also more prevalent in the C2 subtype and high-risk group. Moreover, several immune checkpoint molecules also exhibited higher expressions in the high-risk group, further corroborating the pronounced immunosuppressive TME in this group of HCC patients.

Considering the significantly disparant TME landscapes between the two risk groups, we evaluated the model’s potential utility in predicting immunotherapeutic efficacy. First, the tumor mutation burden was markedly higher in the low-risk group than that in the high-risk group. Moreover, by applying the model to the IMvigor210 tumor ICI treatment cohort, we found that the low-risk group demonstrated more significant post-therapeutic survival improvements. These findings suggest that the low-risk group may gain greater benefit from immunotherapy. For high-risk patients, combining conventional ICI therapies with treatments targeting specific immunosuppressive components in the TME, such as MDSCs and novel immune checkpoint molecules like LAG3, TIGIT, and HAVCR2, may represent a more suitable strategy (7).

The prognostic model developed in this study consists of two genes, TPX2 and TTC26. Encoding a microtubule-associated protein responsible for spindle assembly, TPX2 was overexpressed in many cancers and significantly correlated with high proliferation and aneuploid tumors (26). In HCC, TPX2 overexpression indicates advanced TNM staging and poor tumor differentiation and correlates with unfavorable prognosis (27). Mechanistically, TPX2 may enhance the proliferation, migration, and invasion of HCC cells by activating the PI3K/AKT pathway (28). Additionally, TPX2 can act as a transcriptional coactivator for pregnane X receptor (PXR) and enhance PXR’s binding to the promoter and enhancer regions of the target gene CYP3A4, resulting in accelerated sorafenib clearance in body and leading to sorafenib resistance (29). However, TPX2’s impact on anoikis in malignant tumors has not been reported. TTC26 is a highly conserved protein involved in the generation of intraflagellar transport (IFT) complex, which is a structure essential for the formation and normal function of cilia (30). Dysregulation of TTC26 is closely associated with ciliopathies (31). Nevertheless, the correlation of TTC26 with malignant tumors remains poorly understood. So far, TTC26 was only mentioned in two HCC prognostic models, where high TTC26 expression correlates with higher risk and poorer prognosis (32, 33). In the present study, TTC26 was found to be overexpressed in HCC tumor samples. Besides, TTC26 knockdown significantly impaired the proliferation and migration, while increased the apoptosis of HCC cells, which was indicative of the tumor promoting role of TTC26 in HCC. In addition, these two genes demonstrated certain expression patterns at the single cell and pan-cancer levels, and showed varying degrees of correlations with the therapeutic efficacy of multiple anti-tumor chemotherapeutic and targeted drugs, which would provide novel insights for future studies.

This study has certain limitations. First, it is a retrospective study based on public databases. Although validated in multiple patient cohorts, large-scale prospective studies are still needed to further confirm these findings. Second, while *in vitro* experiments preliminarily identified TTC26 as a novel oncogene in HCC, further in-depth *in vitro* and *in vivo* studies are required to explore their specific pro-tumorigenic mechanisms.

## Data Availability

The original contributions presented in the study are included in the article/supplementary material. Further inquiries can be directed to the corresponding authors.
